# Supplementary material for: DUSP1 regulates apoptosis and cell migration, but not the JIP1-protected cytokine response, during Respiratory Syncytial Virus and Sendai Virus infection
Source: Sci Rep. 2017 Dec 12;7:17388. doi: 10.1038/s41598-017-17689-0 (PMC5727028; doi:10.1038/s41598-017-17689-0)
Supplement: Supplementary file 1 — Supplementary information [file 41598_2017_17689_MOESM1_ESM.pdf]

DUSP1 regulates apoptosis and cell migration, but not the JIP1-protected cytokine response, during Respiratory Syncytial Virus and Sendai Virus infection.

Alexa C. Robitaille[1,2], Elise Caron[1], Nicolas Zucchini[1,3], Espérance Mukawera[1], Damien Adam[1,4], Mélissa K. Mariani[1], Anaïs Gélinas[1,3], Audray Fortin[1], Emmanuelle Brochiero[1,4] and Nathalie Grandvaux[1, 2 *]

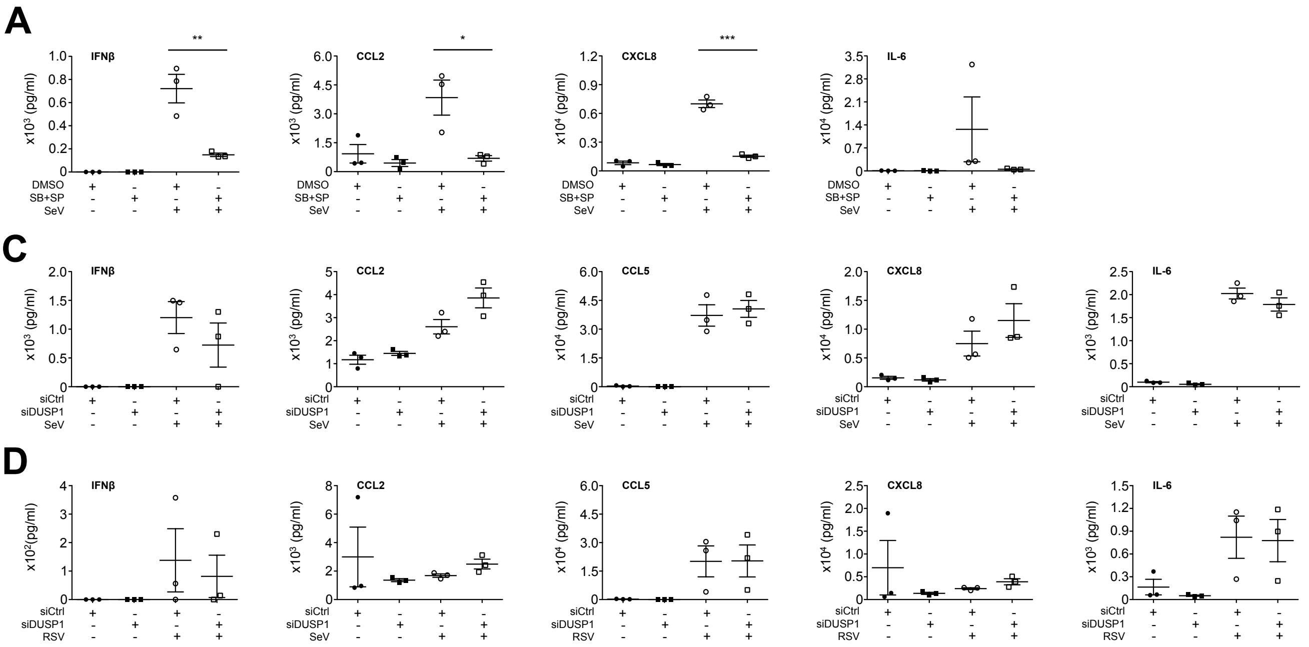

Figure S1: Cytokine production elicited during SeV and RSV infection is protected from DUSP1-mediated inhibition of JNK and p38.

A549 cells were either **(A)** pre-treated with DMSO (vehicle) or SB203580 (10 μM) + SP600125 (10 μM) for 30 min prior to infection**, (B)** transfected with empty or DUSP1-expressing plasmids or **(C and D)** transfected with Control (Ctrl) or DUSP1-specific siRNA before infection with SeV at 40 HAU/10^6 cells **(A to C)** or RSV at MOI of 3 **(D)**. Release of cytokines measured in each biological replicates was quantified using Luminex-based multiplex assays. Data are represented as mean +/- SEM of n≥3 independent replicates and statistical analyzes were done using unpaired t-test. P<0.05 (*), P<0.01 (**) or P<0.001 (***).

B

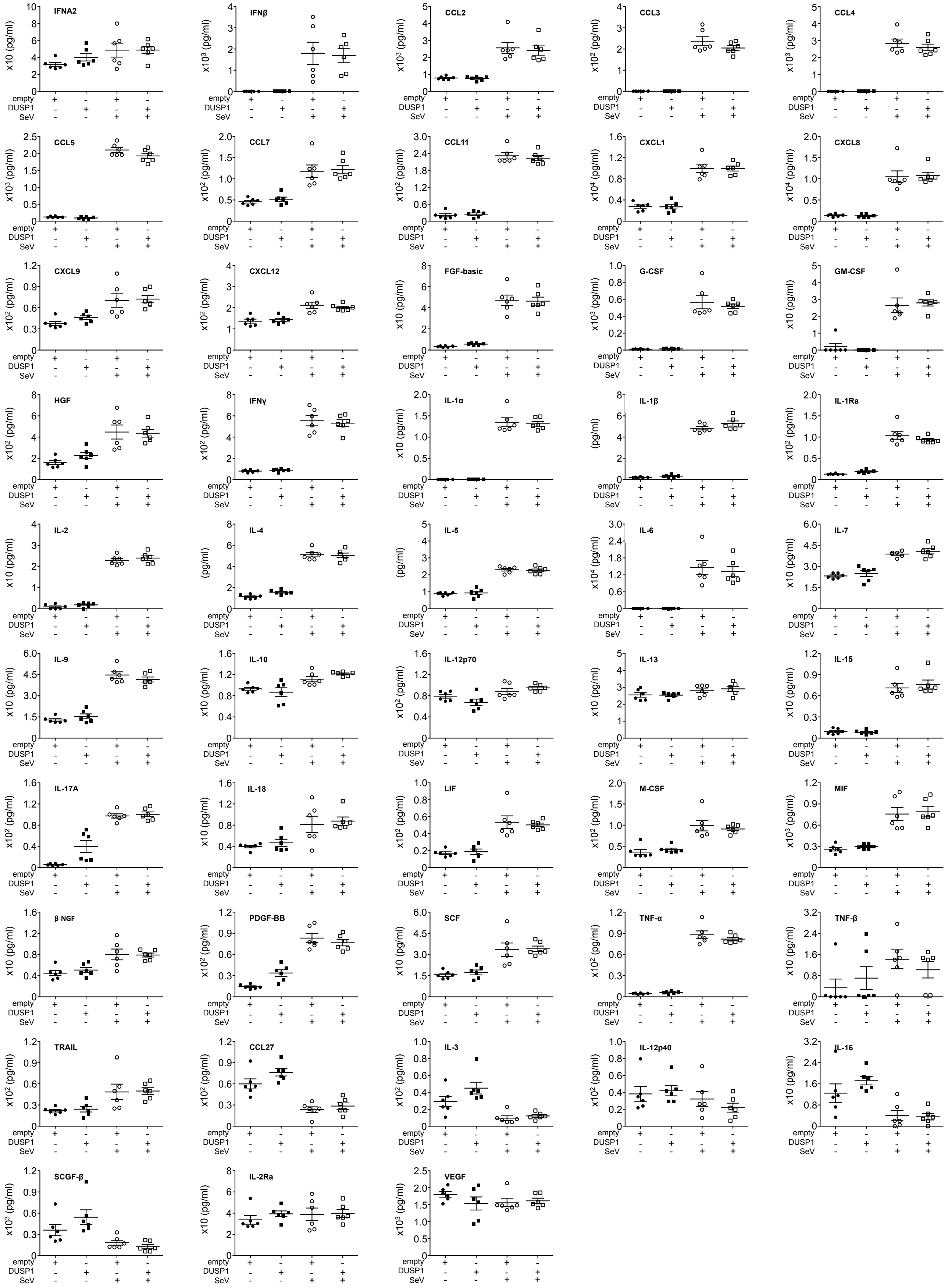
IFNA2
IFNβ
CCL2
CCL3
CCL4
CCL5
CCL7
CCL11
CXCL1
CXCL8
CXCL9
CXCL12
FGF-basic
G-CSF
GM-CSF
HGF
IFNγ
IL-1α
IL-1β
IL-1Ra
IL-2
IL-4
IL-5
IL-6
IL-7
IL-9
IL-10
IL-12p70
IL-13
IL-15
IL-17A
IL-18
LIF
M-CSF
MIF
β-NGF
PDGF-BB
SCF
TNF-α
TNF-β
TRAIL
CCL27
IL-3
IL-12p40
IL-16
SCGF-β
IL-2Ra
VEGF
x10 (pg/ml)
x10³ (pg/ml)
x10² (pg/ml)
x10⁴ (pg/ml)
(pg/ml)
empty
DUSP1
SeV

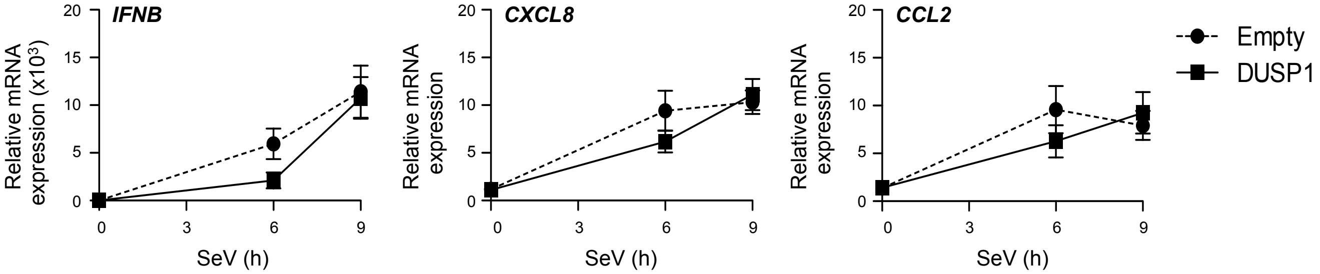

Figure S2: DUSP1 expression does not alter cytokines mRNA levels induced by SeV infection.

A549 cells were transfected with an empty- or DUSP1-expressing plasmid before infection with SeV at 5 HAU/10^6 cells for the indicated times. IFNβ, CXCL8 and CCL2 mRNA levels were quantified by qRT-PCR. Data are represented as mean +/- SEM of n=3 independent experiments and analyzed using two-way ANOVA with Bonferroni's post-test.

Fig. 1A

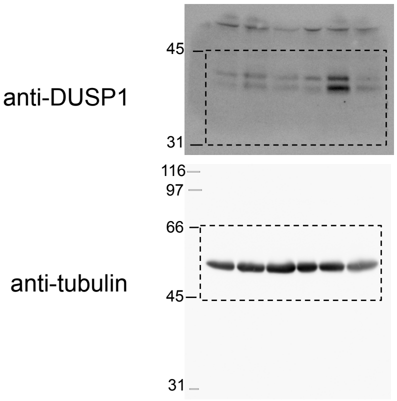

Fig. 1B

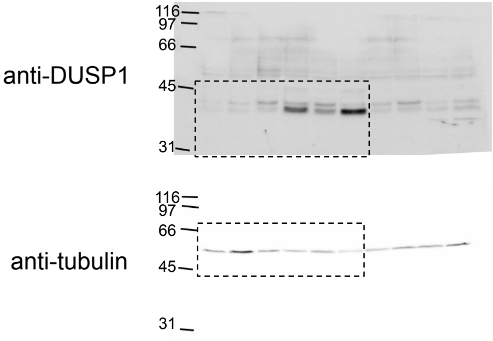

Figure S3: Full-length blots of Figure 1.

Fig. 2A

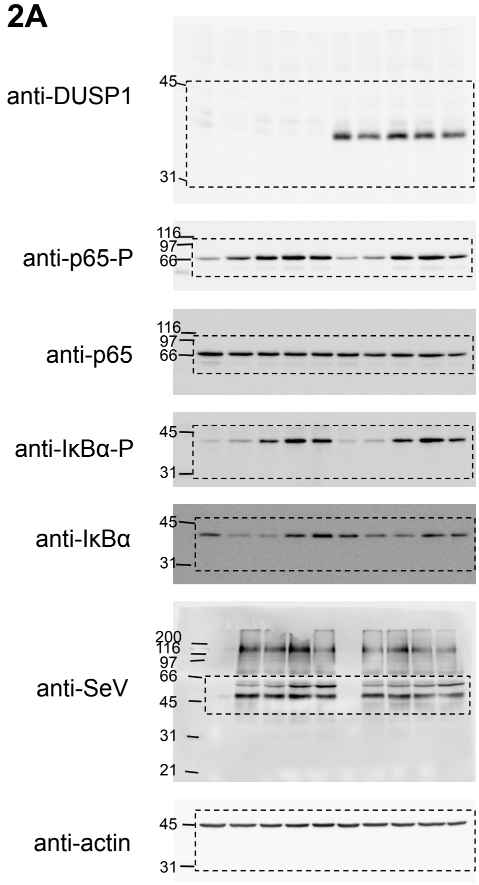

Fig. 2B

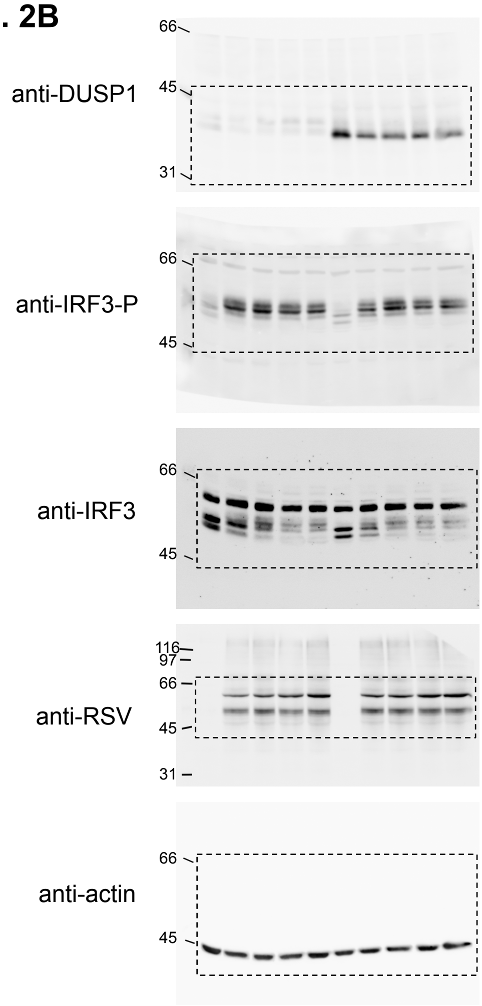

Fig. 2C

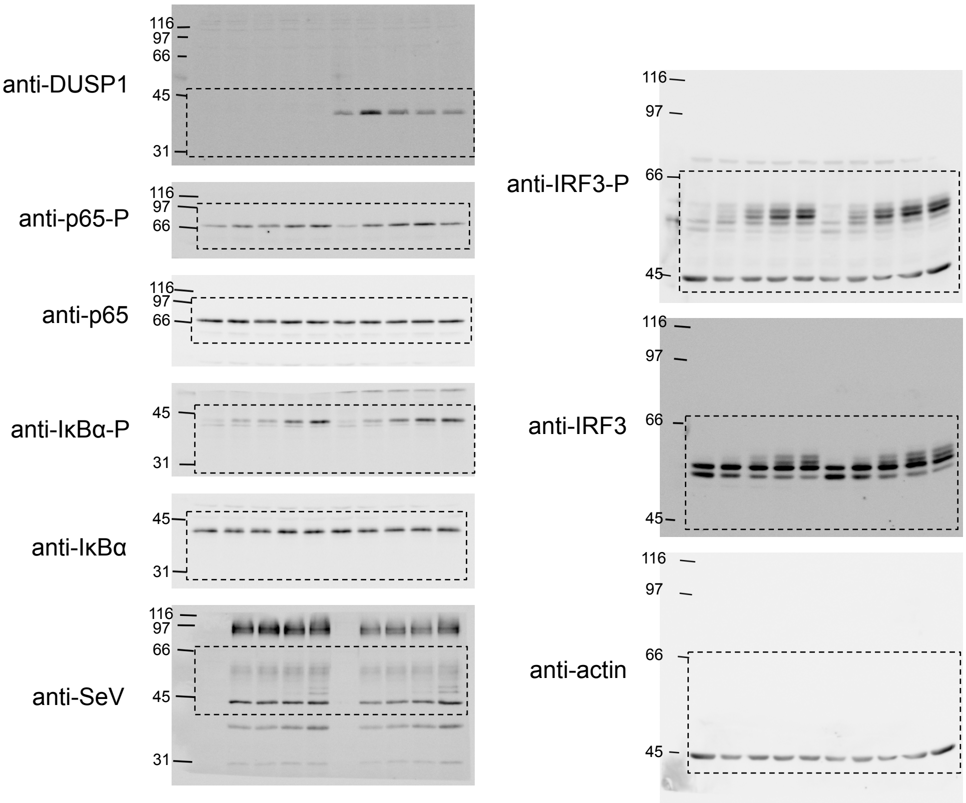

Fig. 2D

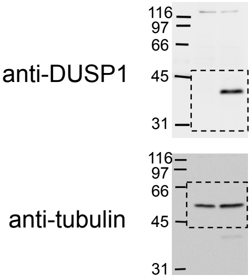

Fig. 2E

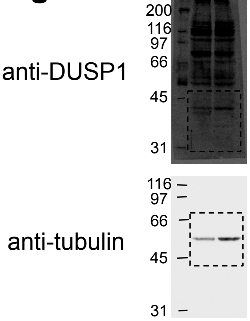

Figure S4: Full-length blots of Figure 2.

Fig. 3A

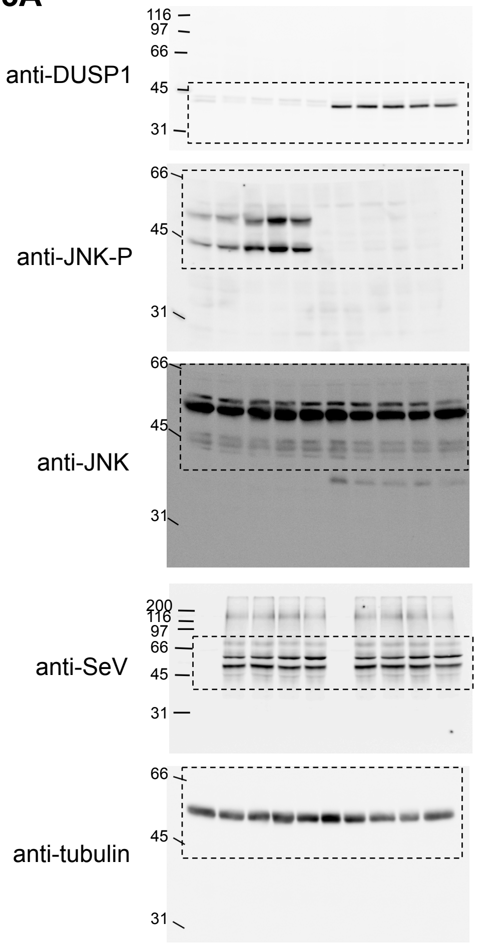

Fig. 3B

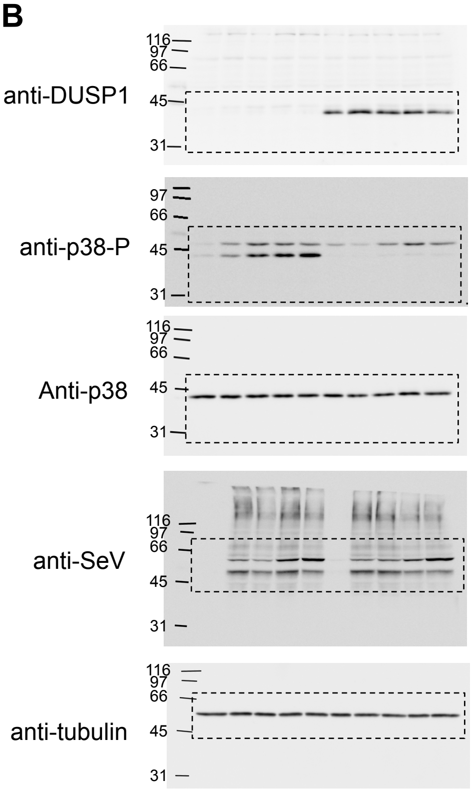

Fig. 3C

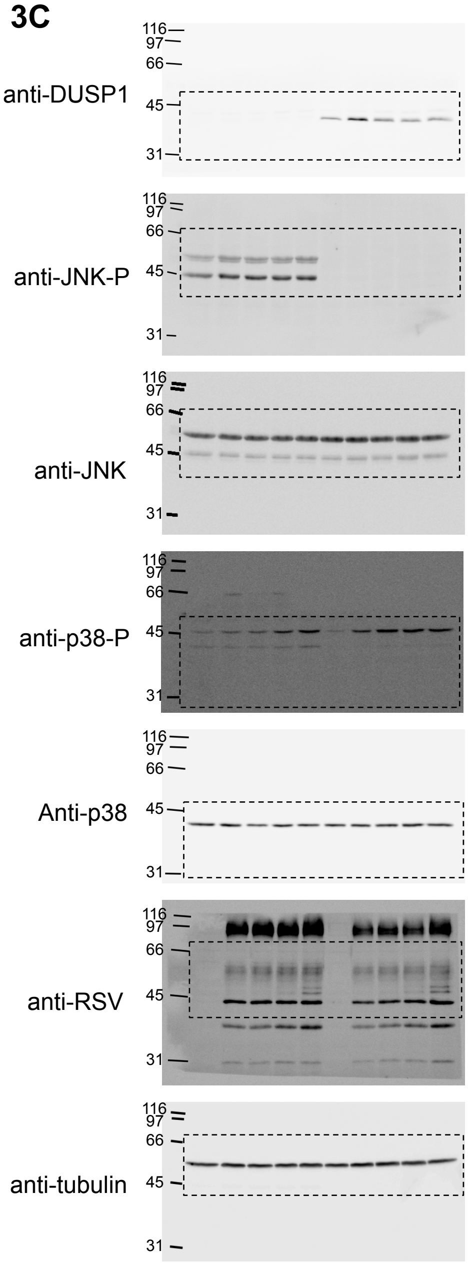

Figure S5: Full-length blots of Figure 3.

Fig. 4A

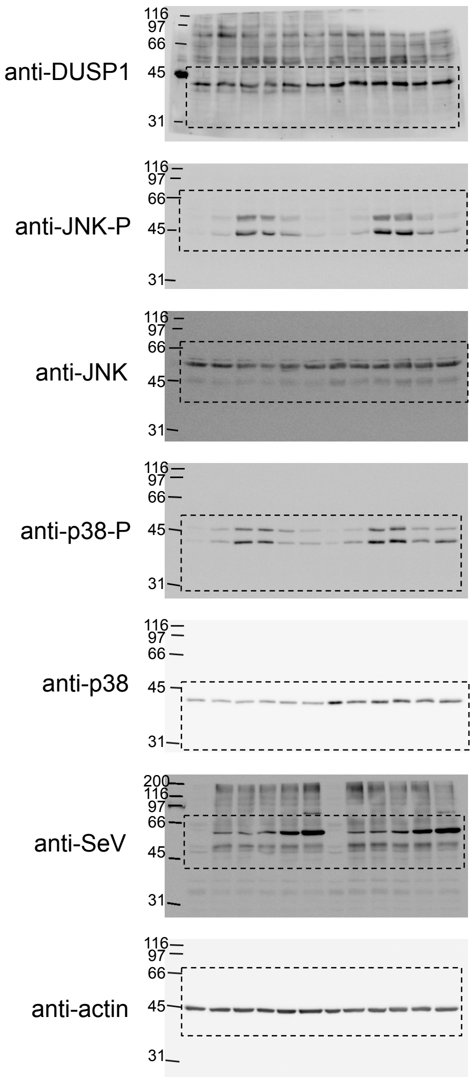

Fig. 4B

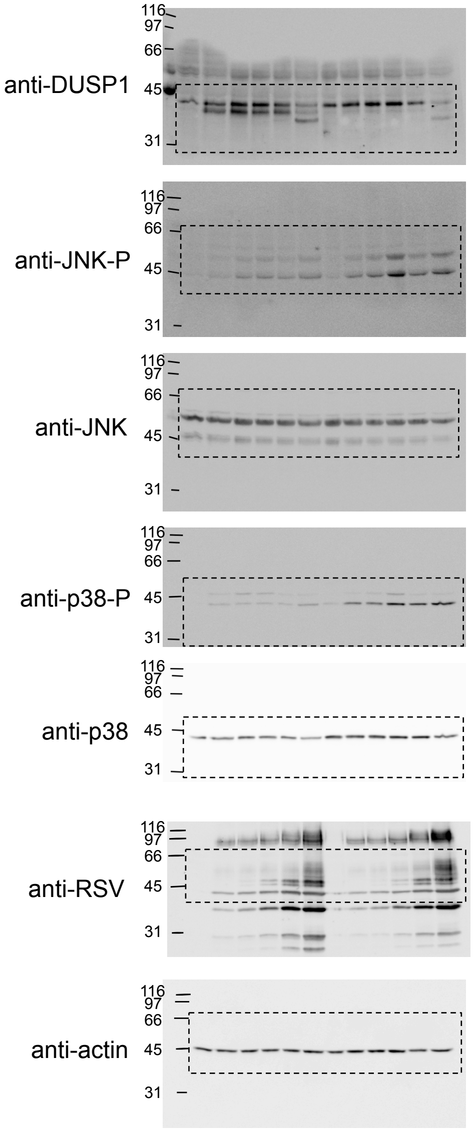

Figure S6 Full-length blots of Figure 4.

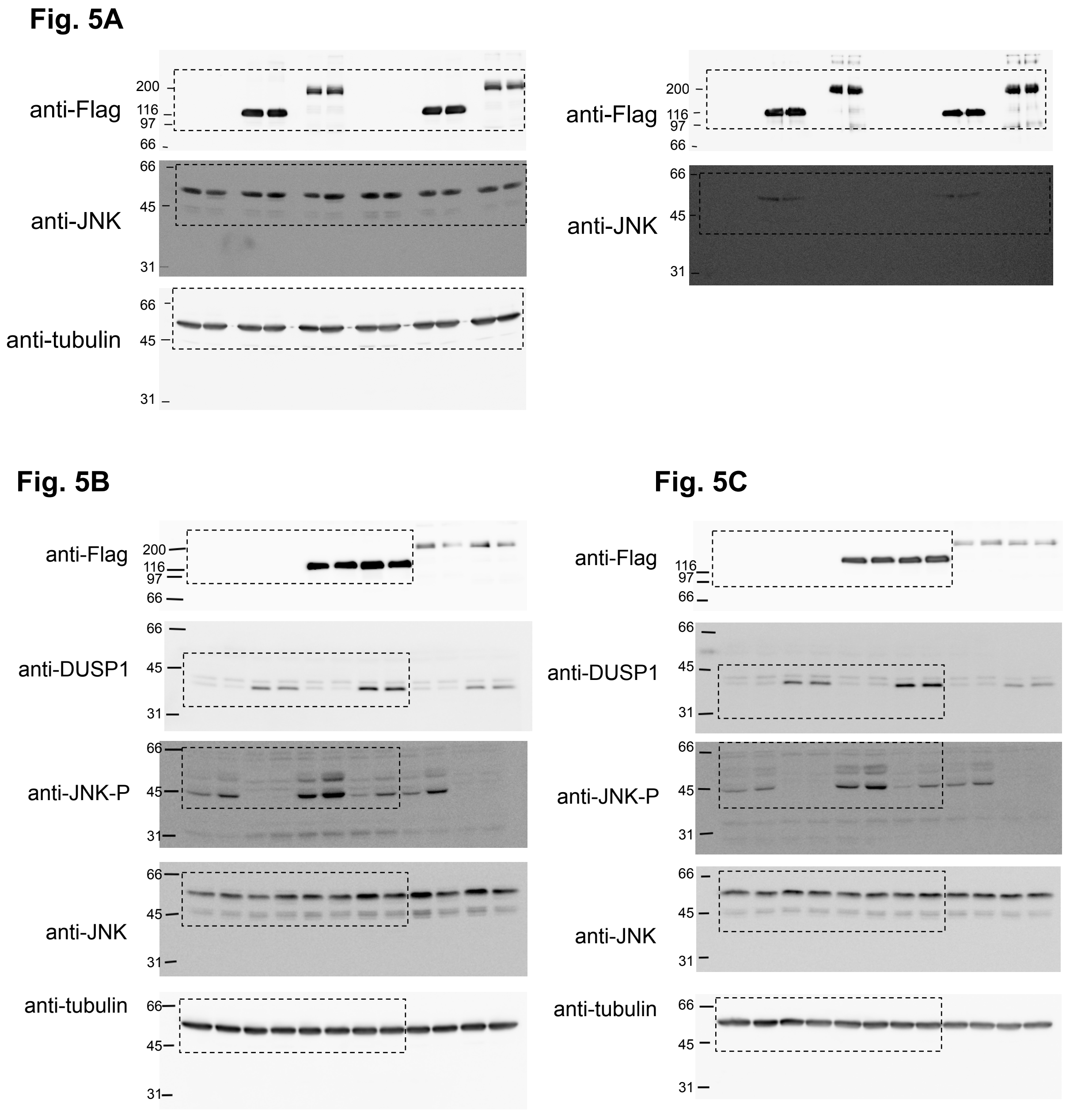

Figure S7: Full-length blots of Figure 5.

Fig. 6A

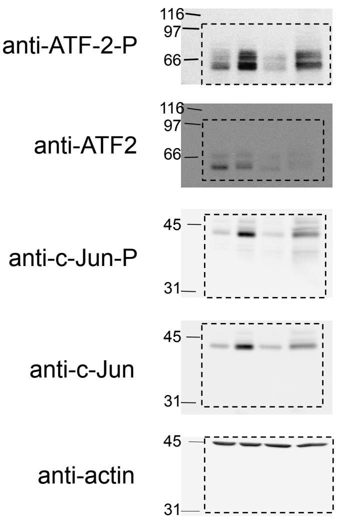

Fig. 6B

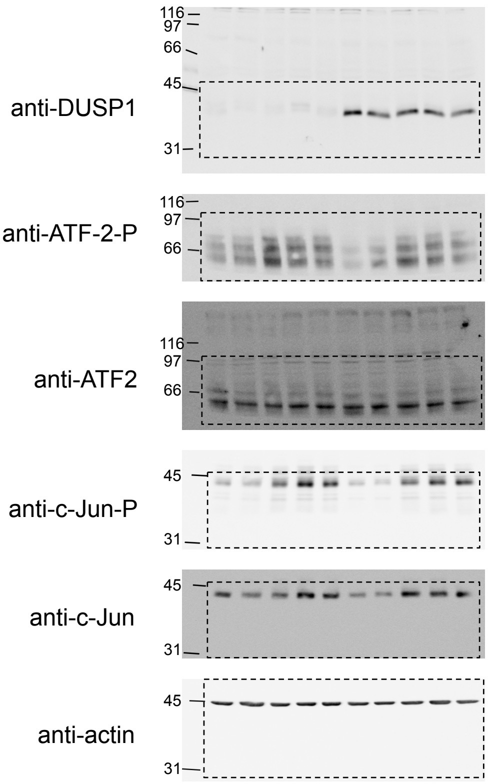

Fig. 6C

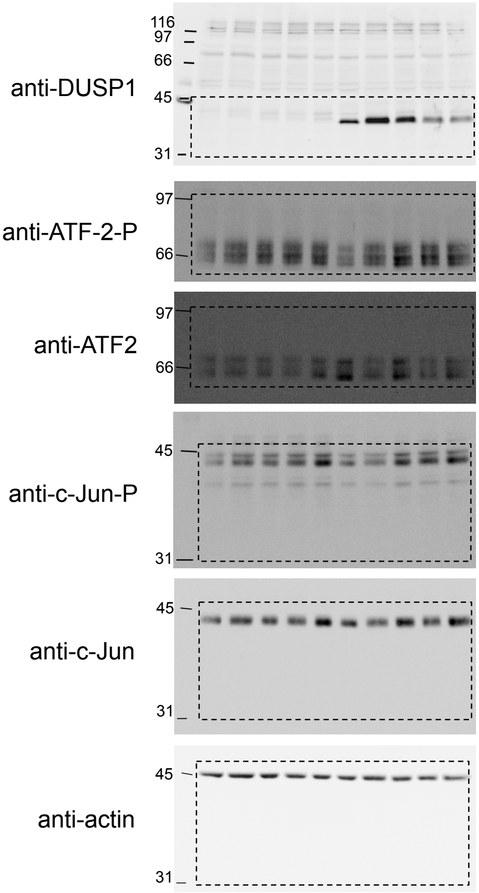

Figure S8: Full-length blots of Figure 6.

Fig. 7

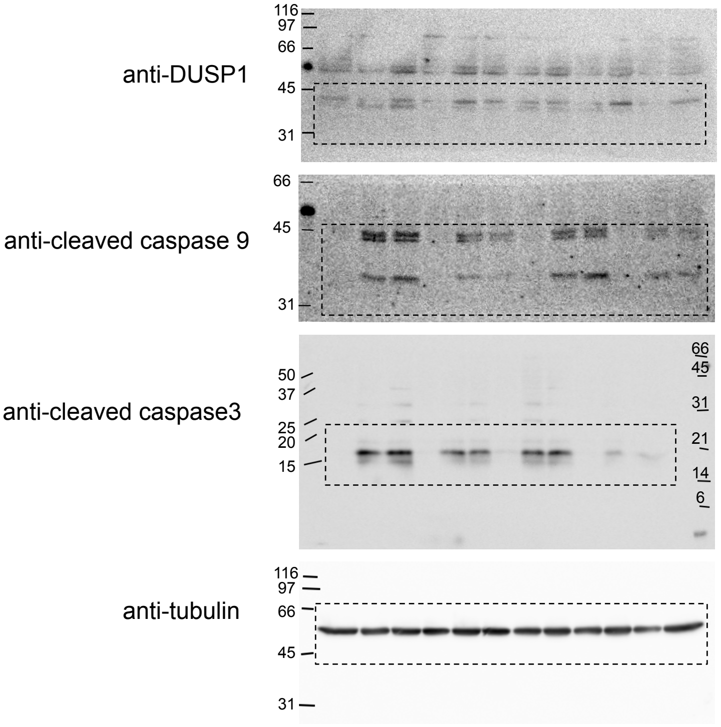

Figure S9: Full-length blots of Figure 7.

SUPPLEMENTARY INFORMATION

MATERIALS AND METHODS

qRT-PCR

Specific mRNA levels were quantified by qRT-PCR as described in the Material and Methods using either Fast start SYBR Green Kit (Roche) for *IFNB* (S: gaactttgacatccctgaggagattaagcagc, AS: gttccttaggatttccactctgactatggtcc) and *IL8* (S: tctcttggcagccttcctgatttc, AS: gtgtggtccactctcaatcactct) or using TaqMan Gene Expression Assays (Applied biosystems) for *CCL2* (#Hs00234140_m1). Results were analyzed by the ΔΔCT method after normalization to S9 mRNA levels (S: cgtctcgaccaagagctga, AS: ggtccttctcatcaagcgtc) or *S9* (Hs02339424_g1) for *CCL2*.
